# How thinking hurts: Rumination, worry, and avoidance processes in adjustment to bereavement

**DOI:** 10.1002/cpp.2440

**Published:** 2020-03-04

**Authors:** Maarten C. Eisma, Thomas A. de Lang, Paul A. Boelen

**Affiliations:** ^1^ Department of Clinical Psychology and Experimental Psychopathology, Faculty of Behavioral and Social Sciences University of Groningen Groningen The Netherlands; ^2^ Department of Clinical Psychology, Faculty of Social Sciences Utrecht University Utrecht The Netherlands; ^3^ ARQ National Psychotrauma Centre Diemen The Netherlands

**Keywords:** anxious avoidance, complicated grief, depressive avoidance, loss avoidance, perseverative cognition, persistent complex bereavement disorder

## Abstract

Repetitive negative thought plays an important role in the maintenance of mental health problems following bereavement. To date, bereavement researchers have primarily focused on rumination (i.e., repetitive thought about negative events and/or negative emotions), yet the interest in worry (i.e., repetitive thought about uncertain future events) is increasing. Both cognitive processes potentially lead to poorer adaptation to bereavement by contributing to loss‐related avoidance and behavioural avoidance of activities. The current study aims to establish the differential associations of rumination and worry with symptoms of depression and prolonged grief and clarify if avoidance processes mediate the associations of rumination and worry with symptom levels. Four hundred seventy‐four recently bereaved adults (82% female) filled out questionnaires assessing rumination, worry, loss‐related and behavioural avoidance, and depression and prolonged grief symptoms. Rumination and worry were both uniquely associated with depression and prolonged grief symptoms. Compared with worry, rumination related more strongly to prolonged grief symptoms, whereas correlations of both cognitive styles with depression symptoms did not differ. Loss‐related avoidance and behavioural avoidance partially mediated the associations of rumination and worry with prolonged grief symptoms. Behavioural avoidance partially mediated the associations of rumination and worry with depression symptoms. Findings suggest that exposure and behavioural activation may be effective interventions to reduce repetitive thinking and psychopathology after bereavement.

**Key Practitioner Message**

- Worry and rumination are both uniquely associated with post‐loss mental health problems.
- Loss avoidance and behavioural avoidance mediate the relationships of worry and rumination with prolonged grief symptoms.
- Behavioural avoidance mediates the relationships of worry and rumination with depression symptoms.
- Findings suggest exposure therapy, and behavioural activation may be effective in reducing post‐loss repetitive thought and psychopathology.

## 1 INTRODUCTION

Repetitive thought, the process of thinking attentively, repetitively, or frequently about one's self and one's world (Segerstrom, Stanton, Alden, & Shortridge, 2003), has been related to poorer mental health after bereavement (Nolen‐Hoeksema, 2001; Watkins & Moulds, 2013). Repetitive thought has drawn considerable research interest, as it is amenable to change in therapy (Querstret & Cropley, 2013; Spinhoven et al., 2018). Therefore, enhancing understanding of this cognitive process may improve treatments for people who experience severe mental health problems after bereavement.

The current study was designed to shed light on the effects and working mechanisms of two oft‐studied repetitive thought styles, rumination and worry, in adaptation to loss. Conceptually, rumination and worry share certain characteristics; they are both abstract, verbal thinking styles, and transdiagnostic risk factors for mental health problems (for reviews, see Aldao & Nolen‐Hoeksema, 2010; Watkins, 2008). However, they are also conceptually distinct phenomena, with key differences in thought content, temporal orientation, and motivation. Whereas rumination is focused on past negative events and affect, with the primary motive to better understand one's inner world, worry is focused on future uncertain events with potential negative outcomes, with a primary motive to prevent bad things from happening (for comparisons, see Nolen‐Hoeksema, Wisco, & Lyubomirsky, 2008; Pillai & Drake, 2015). Factor analyses, latent profile analyses, and structural equation modelling have provided evidence both for a single underlying factor to worry and rumination, that is, repetitive negative thought, and for the distinct properties of each construct (e.g., Muris, Roelofs, Meesters, & Boomsma, 2004; Segerstrom, Tsao, Alden, & Craske, 2000; Siegle, Moore, & Thase, 2004; Wisco, Plate, May, & Aldao, 2018).

In the context of bereavement, most research attention has focused on rumination, whereas studies on worry are scarce. Nevertheless, there is growing evidence that both forms of repetitive thinking impede psychological recovery after loss. Whereas rumination can be interpreted as an attempt to understand the death and related negative emotions, worry is regarded as an effort to manage the secondary stressors that often arise in the aftermath of a loss (e.g., poor coping of family members, relationship difficulties, and taking on new roles and responsibilities; Eisma, Boelen, Schut, & Stroebe, 2017). Rumination has been found to be concurrently and longitudinally associated with anxiety, depression, post‐traumatic stress, and prolonged grief symptoms (e.g., Morina, 2011; Nolen‐Hoeksema, Parker, & Larson, 1994; Smith & Ehlers, in press; for a recent review, see Eisma & Stroebe, 2017). Similarly, three survey studies have demonstrated worry to be concurrently and longitudinally associated with anxiety, depression, and prolonged grief severity (Boelen, 2010; Boelen, Reijntjes, & Smid, 2016; Eisma et al., 2017).

To date, there has been only one empirical study simultaneously examining the effects of worry and rumination on adaptation to bereavement, suggesting that rumination was more strongly associated with post‐loss psychopathology than worry (Boelen et al., 2016). Outside the bereavement area, comparisons of the effects of rumination and worry have yielded mixed findings. For instance, one longitudinal study in a nonclinical student sample demonstrated that worry was related to both depression and anxiety symptoms whereas rumination was only related to depression symptoms (Hong, 2007). Another longitudinal survey in a similar sample showed that rumination and worry both relate to anxiety symptoms but not depression symptoms (Calmes & Roberts, 2007). Clarifying the relative contribution of worry and rumination to post‐loss mental health problems is important, as it offers clues on what repetitive thought style(s) should be targeted in prolonged grief treatments. For example, within metacognitive therapy, therapists may be advised to focus on changing positive and negative beliefs related to either rumination or worry, dependent on which of these processes is most strongly connected to emotional distress following loss (Wenn, O'Connor, Kane, Rees, & Breen, 2019).

Despite their conceptual differences and potentially divergent effects on mental health, rumination and worry may perpetuate mental health problems after bereavement in similar ways. Specifically, although early rumination researchers considered rumination to be a coping style that is similar to confrontation (Nolen‐Hoeksema, 2001; Nolen‐Hoeksema & Larson, 1999), more recent research has suggested that rumination may serve as a type of cognitive avoidance of emotionally laden loss‐related material. For example, Eisma et al. (2013) provided evidence that the chronic abstract, verbal thoughts during rumination may serve to suppress more threatening loss‐related thoughts. Broadly speaking, “loss‐related avoidance” (also termed “anxious avoidance”) refers to the avoidance of cues connected with the irreversibility of the loss, driven by the fear that confronting this irreversibility is “unbearable” (Boelen, van den Hout, & van den Bout, 2006). This loss‐related avoidance, in turn, could hamper integration of the reality of the loss with existing autobiographical memory about the self, the lost person, and the future, thereby perpetuating loss‐related distress (Boelen et al., 2006; cf. Stroebe et al., 2007; Worden, 2009). Indeed, survey research in nonbereaved and bereaved samples generally confirm associations between rumination, cognitive avoidance (including loss‐related avoidance), and mental health problems (e.g., Eisma et al., 2013; Morina, 2011; Moulds, Kandris, Starr, & Wong, 2007; Wenzlaff & Luxton, 2003). Rumination has also been found to be associated with automatic and voluntary avoidance of loss‐reality cues in two laboratory studies (Eisma et al., 2014; Eisma et al., 2015). Moreover, a randomized controlled trial demonstrated that reducing avoidance of loss‐related stimuli through exposure yielded reductions in rumination and loss‐related psychopathology (Eisma et al., 2015).

Worry researchers have similarly argued that worry could serve as cognitive and/or emotional avoidance (Borkovec & Ray, 1998; cf. Newman & Llera, 2011). As such, it has been posited that worry—as an abstract, verbal thinking pattern similar to rumination—could serve to suppress threatening loss‐related thoughts, thereby maintaining acute loss‐related distress (Eisma et al., 2017). Indeed, research outside the bereavement area has shown positive associations between worry and cognitive and experiential avoidance (e.g., Bird, Mansell, Dickens, & Tai, 2013; for a meta‐analysis, see Naragon‐Gainey, McMahon, & Chacko, 2017). Moreover, exposure‐based treatments for anxiety‐related disorders effectively reduce worry (e.g., Goldman, Dugas, Sexton, & Gervais, 2007; Hoyer et al., 2009).

From a theoretical viewpoint, worry and rumination may also exacerbate post‐loss mental health through a conceptually different avoidance process. Building on theories about behavioural activation (Ferster, 1973; Martell, Addis, & Jacobson, 2001), Nolen‐Hoeksema et al. (2008) proposed a link between rumination and the tendency to withdraw from social, occupational, and recreational activities. This process has been termed “behavioural avoidance” (or, in the context of grief, “depressive avoidance,” Boelen et al., 2006). Nolen‐Hoeksema et al. (2008) proposed that rumination helps individuals to avoid an aversive environment because it occupies attention and time. Moreover, rumination could serve to build a case that one is facing a hopelessly uncontrollable situation and that nothing can be done to overcome it. Rumination as such not only removes people from aversive situations but also provides them with reasons to withdraw from such situations. In turn, decreased participation in activities could reduce access to experiences that could refute negative beliefs thereby fuelling negative affect and, ultimately, depression (Martell et al., 2001). Behavioural withdrawal may also prevent bereaved people from engaging in experiences in which the absence of the deceased is felt strongly and, consequently, interfere with the integration of the loss into autobiographical knowledge about the self and the lost person, thereby maintaining acute grief responses (e.g., Boelen et al., 2006). Similar to rumination, worry could take up attention and time, thereby reducing activity levels. Furthermore, as worry increases perceptions of the probability and costs of potentially negative undesirable outcomes for future events (Berenbaum, Thompson, & Bredemeier, 2007), it may provide yet another rationale for behavioural withdrawal after bereavement.

In line with the above, associations between rumination and behavioural avoidance have been found in surveys in nonbereaved and bereaved samples (e.g., Eisma et al., 2013; Moulds et al., 2007; for a meta‐analysis, see Naragon‐Gainey et al., 2017). Moreover, behavioural avoidance and post‐loss mental health problems are strongly associated (e.g., Boelen & Eisma, 2015; Boelen & Van den Bout, 2010; Monk, Houck, & Katherine Shear, 2006; Stahl & Schulz, 2018), and behavioural activation reduced prolonged grief and rumination in two randomized controlled trials (Eisma, Boelen, et al., 2015; Papa, Sewell, Garrison‐Diehn, & Rummel, 2013). Research on worry and behavioural avoidance is yet limited (and non‐existent in bereaved samples), yet Pietrzak, Harpaz‐Rotem, and Southwick (2011) found a positive association between worry, social avoidance, and post‐traumatic stress symptoms in a veteran sample. Chen, Liu, Rapee, and Pillay (2013) further demonstrated in a randomized controlled trial in a community sample that excessive worry can be effectively treated with behavioural activation.

The overarching goal of the current study was to further our understanding of potential working mechanisms of rumination and worry in impeding adaptation to bereavement. The first aim of this study was to establish the relative association of rumination and worry with depression and prolonged grief symptoms. We predicted that rumination and worry would both be positively associated with depression and prolonged grief symptoms. We also predicted that rumination would be more strongly associated with depression and prolonged grief symptoms than worry (cf. Boelen et al., 2016) and that worry would still be associated with depression and prolonged grief symptoms after controlling for effects of rumination and relevant background variables.

The second aim of our study was to clarify the pathways along which rumination and worry contribute to post‐loss mental health problems. We predicted that rumination would exert significant indirect effects on depression and prolonged grief symptoms via both loss‐related avoidance (i.e., “anxious avoidance”) and behavioural avoidance (i.e., “depressive avoidance”). Similarly, we hypothesized that worry would have indirect effects on depression and prolonged grief symptoms via loss‐related avoidance and behavioural avoidance.

## 2 METHODS

### 2.1 Procedure and participants

Data were collected as part of a larger ongoing research programme on the development and cognitive and behavioural correlates of emotional responses following loss (the Utrecht Longitudinal Study on Adjustment to Loss). A local ethical review board approved the study. Participants were recruited via announcements on Internet websites with information about grief. The announcements explained the aims of the research programme and invited bereaved adults (i.e., 18 years and older) to participate. After completing an online application form, participants were sent a personal login code and were referred to a secured website where more information about the study was given. After giving informed consent, participants could complete the questionnaire. Only adults bereaved of a significant other were allowed to participate. No other inclusion or exclusion criteria were applied.

We used data from 474 participants (82% female) who had lost a relative or friend in the last 3 years. The mean age was 54.5 (*SD* = 13.2) years. Most participants experienced the loss of a partner, 45%, or a parent, 37%. The average time since lost was 9.62 (*SD* = 9.20) months. Full sample characteristics are shown in Table 1.

**Table 1:** Demographic and loss‐related characteristics of the sample (*N* = 474)

Demographic variables	
Gender—valid *N* (%)	
Male	86 (18.1)
Female	388 (81.9)
Age in years—*M* (*SD*), range	54.5 (13.2) 18–89
Education level—valid *N* (%)	
College/university	259 (54.6)
Other	215 (45.4)
Loss characteristics	
Time since loss—*M* (*SD*), range	9.6 (9.2) 0–36
Deceased person—valid *N* (%)	
Partner	211 (44.5)
Child	40 (8.4)
Sibling	24 (5.1)
Parent	173 (36.5)
Other	26 (5.5)
Cause of death—valid *N* (%)	
Disease longer than 1 month	225 (47.5)
Disease shorter than 1 month	41 (8.6)
Accident	12 (2.5)
Suicide	25 (5.3)
At birth	4 (0.8)
Unexpected medical cause	97 (20.5)
Other cause	70 (14.8)^a^
Death—valid *N* (%)	
Non‐violent	426 (89.9)
Violent (suicide, accident, and homicide)	48 (10.1)

a Remarks provided classified 11 answers as violent deaths, of which eight were homicides.

### 2.2 Materials

This study is part of a larger research programme, and only instruments relevant for the current study are described here.

#### 2.2.1 Sociodemographic and loss‐related characteristics

A self‐constructed questionnaire was used to assess the sociodemographic characteristics (sex, age, and education level) and loss‐related characteristics (kinship to the deceased, cause of death, and time since loss).

#### 2.2.2 Rumination

To assess the general tendency to engage in maladaptive depressive rumination, we used the brooding subscale of the Ruminative Response Scale (RRS; Dutch version: Schoofs, Hermans, & Raes, 2010; Treynor, Gonzalez, & Nolen‐Hoeksema, 2003). The brooding subscale has been proposed to show no content overlap with depression symptoms (in contrast to the full RRS). Participants were asked to indicate what they typically do when feeling down, blue, or depressed. Five statements on ruminative responses are rated on a 4‐point scale ranging from 1 (*almost never*) to 4 (*almost always*). Higher total scores represent more brooding. In the current study, the brooding scale's internal consistency was acceptable (*α* = .79).

#### 2.2.3 Worry

To assess the general tendency to engage in worry, the abbreviated version of the Penn State Worry Questionnaire (PSWQ‐A; Hopko et al., 2003; Dutch version: Boelen et al., 2016) was administered. The PSWQ‐A contains eight items of worry rated on a 5‐point scale from 1 (*not at all typical of me*) to 5 (*very typical of me*). Higher sum scores indicate stronger worry tendencies. In the present investigation, the reliability of the PSWQ‐A was excellent (*α* = .94).

#### 2.2.4 Loss‐related and behavioural avoidance

Loss‐related and behavioural avoidance were assessed using the Depressive and Anxious Avoidance in Prolonged Grief Questionnaire (DAAPGQ; Boelen & van den Bout, 2010). The DAAPGQ was specially developed to measure two distinct processes related to prolonged grief. Anxious avoidance (i.e., loss‐related avoidance), tapped with four items, measures cognitive avoidance of painful aspects of the loss (e.g., “I avoid to dwell on painful thoughts and memories connected to his/her death”). Depressive avoidance (i.e., behavioural avoidance), assessed with five items, measures behavioural avoidance of activities (e.g., “Since [–] died, I avoid activities that used to give me satisfaction, because these activities now seem meaningless to me”). Items are rated on an 8‐point scale ranging from 1 (*Not at all true for me*) to 8 (*Completely true for me*). In the current sample, internal consistencies of the anxious avoidance and depressive avoidance subscales were excellent (*α* = .92) and acceptable (*α* = .77), respectively.

#### 2.2.5 Prolonged grief symptoms

Prolonged grief symptoms were measured using the 11‐item Prolonged Grief Disorder Scale (PGD Scale: Boelen, Keijsers, & van den Hout, 2012). This scale follows the diagnostic suggestions of Prigerson et al. (2009) for PGD. It contains items on cognitive/emotional symptoms, separation distress, and functional impairment. The items are based on the Inventory of Complicated Grief—Revised (Prigerson & Jacobs, 2001) and are rated on a 5‐point scale ranging from 1 (*never*) to 5 (*always*). The sum score represents PGD symptom severity. Internal consistency for the current sample was excellent (*α* = .91).

#### 2.2.6 Depression symptoms

To assess depression symptoms, the depression subscale of the Hospital Anxiety and Depression Scale (HADS) was administered (Zigmond & Snaith, 1983; Dutch version: Spinhoven et al., 1997). Participants rated on seven items to what extent they experienced certain symptoms in the last week. Answers were given on a 4‐point scale ranging from 0 to 3 (varying anchors). The depression subscale of the HADS has good psychometric properties (Bjelland, Dahl, Haug, & Neckelmann, 2002). In the current study, internal consistency was excellent (*α* = .93).

### 2.3 Statistical analyses

All analyses were conducted in SPSS Version 25 (IBM Corporation, 2017). First, we recoded the demographic variables “cause of death” into non‐violent and violent (the latter category consisting of suicides, accidents, and homicides). Next, using independent *t* tests, ANOVAs, and correlations, we evaluated the relationships between all demographic and loss‐related variables and the dependent variables, depression and prolonged grief symptoms. Variables that significantly related to the dependent variables were included as control variables in all main analyses.

Next, zero‐order correlations were calculated to assess relationships between the independent variables (worry and rumination), mediators (loss‐related avoidance and behavioural avoidance), and dependent variables (symptom levels of depression and prolonged grief). To compare differential associations of rumination and worry with depression and prolonged grief symptoms, we first tested whether the correlations between rumination and prolonged grief and depression symptoms were significantly different from worry with both symptom measures, using the dependent‐sample correlation comparison *z*‐test developed by Lenhard and Lenhard (2014).

Before conducting the main analyses, we checked the assumptions of regression analyses. We inspected scatterplots, normal probability plots, and residual plots to check assumptions of linearity, normality of the residuals, and homoscedasticity, respectively, for the relations between rumination, worry, loss‐related and behavioural avoidance, and prolonged grief and depression symptoms. Next, two separate hierarchical regression analyses were performed with depression and prolonged grief severity as dependent variables, including relevant background variables as independent variables in the first step of the model, rumination in the second step of the model, and worry in the third step of the model.

**Figure 1:**
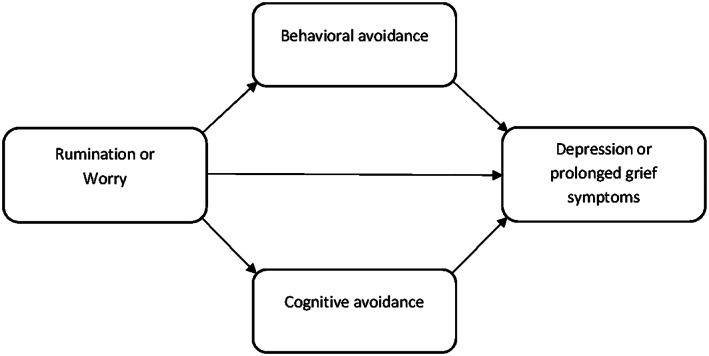
Graphical display of the proposed mediation models

Lastly, four multiple mediation models were run (Figure 1) using the PROCESS macro of SPSS (v3.2, Model 4; Hayes, 2013). Two mediation models included rumination as an independent variable and depression and prolonged grief symptoms as dependent variables. Two additional mediation models included worry instead of rumination as an independent variable. Of primary interest to our study were the indirect effects of the independent variables (worry and rumination), via the two mediators (loss‐related and behavioural avoidance), on the dependent variables (prolonged grief and depression symptoms; path a × path b). We determined the direct effect of each independent variable on each dependent variable (path c) and the direct effect that remained when controlling for the effects of the mediators (path c′). The relative difference between the original direct effect (path c) and the remaining direct effect (path c′) was used as an indicator of the effect size of the mediation using the formula suggested by MacKinnon, Fairchild, and Fritz (2007), 1 − c′/c (outcome range between 0 and 1). Bootstrapping with 5,000 resamples was used to test whether effects were significant, using a 95% confidence interval (CI). Standardized effect sizes are reported to facilitate straightforward comparisons between models. Standardized variables were used to derive *β* estimates. Less than 4% of item on dependent and independent variables were missing. Missing items where imputed by the item mean score of the respective scale. Analyses without imputed values yielded nearly identical results to the analyses described below.

## 3 RESULTS

### 3.1 Preliminary analyses

From our sample, 28 (6%) met criteria for probable PGD, as evidenced by a loss experienced more than 5 months previously (i.e., time since loss ≥6 months), a score of 4 or higher on the yearning and functional impairment items of the PGD scale, and at least five additional symptoms (cf. Prigerson et al., 2009). Furthermore, 37% scored higher than 7 on the depression subscale of the HADS, a cut‐off point for clinically relevant depression symptoms (Bjelland et al., 2002).

Higher depression symptom levels were experienced by participants who were female, *t*(472) = 2.83, *p* = .005, younger, *r*(472) = −.21, *p* < .001, experienced a violent loss, *t*(472) = 3.50, *p* = .001, or lost a partner or child, *F*(4, 469) = 8.08, *p* < .001 (all post hoc analyses comparisons *ps* < .01—except for sibling loss). Therefore, sex, age, cause of death, and kinship were controlled for in the regression and mediation models using depression symptoms as a dependent variable.

Higher prolonged grief symptoms were found for females, *t*(472) = 3.41, *p* = .001, and people who were younger, *r*(472) = −.22, *p* < .001, lower educated, *t*(472) = 2.91, *p* = .004, experienced violent loss, *t*(472) = 4.06, *p* < .001, or lost a partner or child, *F*(4, 469) = 7.39, *p* < .001 (all post hoc analyses comparisons *ps* < .01—except for sibling loss). Hence, sex, age, education level, cause of death, and kinship were controlled for in the regression and mediation models with prolonged grief symptoms as dependent variable.

Assumption checks showed that some of the associations of independent variables with depression symptoms, minor violations of normality of errors, and homoscedasticity were present. The central limit theorem implies that our large sample protects our analysis from errors associated with violations of the normality assumption (Ernst & Albers, 2017). Heteroscedasticity in some relations was also unproblematic, as estimations will still be unbiased and consistent if the variance is finite (Chatterjee & Hadi, 2006).

### 3.2 Correlations between rumination, worry, avoidance processes, and depression and prolonged grief symptoms

Rumination, worry, loss‐related and behavioural avoidance, and depression and prolonged grief symptoms were all significantly and positively correlated (Table 2). Correlations of worry and rumination with depression severity did not differ significantly (*Z* = 0.85, *p* = .20). Correlations of rumination and worry with prolonged grief severity, however, were significantly different (*Z* = 2.64, *p* = .008), with rumination being more strongly related to prolonged grief symptoms than worry.

**Table 2:** Correlations between independent, mediator, and outcome variables (*N* = 474)

	Worry	Loss‐related avoidance	Behavioural avoidance	Depression symptoms	Prolonged grief symptoms	*M*	*SD*
Rumination	.61*	.41*	.45*	.47*	.54*	8.95	3.11
Worry	—	.34*	.41*	.44*	.45*	20.3	8.25
Loss‐related avoidance		—	.50*	.39*	.55*	11.4	6.61
Behavioural avoidance			—	.82*	.75*	15.5	9.89
Depression symptoms				—	.77*	6.03	5.10
Prolonged grief symptoms					—	27.9	9.70

* *p* < .001.

### 3.3 Regression analyses of rumination and worry on depression and prolonged grief symptoms

The hierarchical regression analyses are summarized in Table 3. The first model, in which depressive symptoms were the dependent variable, was significant, *F*(9, 464) = 29.48, *p* < .001. The first step, with sociodemographic and loss‐related variables included as independent variables, explained 16% of the variance in depression symptoms. Of the background variables, only kinship and age added significantly to the final model for depression symptoms. The addition of rumination in the second step explained another 17% of variance (*β* = .28, *p* <. 001). Worry, added in the last step, explained an additional 4% of variance in depression symptoms (*β* = .26, *p <.* 001).

**Table 3:** Hierarchical regression of rumination and worry on depression and prolonged grief symptoms

	Depression symptoms			Prolonged grief symptoms		
	*ΔF* (*df*)	*ΔR* ^2^	*β*	*ΔF* (*df*)	*ΔR* ^2^	*β*
Step 1	12.50 (7,466)	.16***		13.10 (8,465)	.18***	
Sex			.03			.05
Age			−.12**			−.11**
Education			—			−.03
Kinship 1			.35***			.43***
Kinship 2			.22***			.23***
Kinship 3			.12			.16**
Kinship 4			.04*			.15
Cause death			.06			.10
Step 2	114.44 (1,465)	.17***		164.20 (1,464)	.21***	
Rumination			.28***			.38***
Step 3	28.73 (1,464)	.04***		18.84 (1,463)	.02**	
Worry			.26***			.20***

*Note.* Reported *β*s are from final model only. Kinship is dummy coded: kinship 1 = partner vs. other, kinship 2 = child vs. other, kinship 3 = sibling vs. other, kinship 4 = parent vs. other. Education is dummy coded: college/university vs. other. Cause of death is dummy coded: violent vs. non‐violent.

* *p* < .05.

** *p* < .01.

*** *p* < .001.

The second model, in which prolonged grief symptoms was the dependent variable, was also significant, *F*(10, 463) = 33.64, *p* < .001. In the first step, relevant demographic and loss‐related variables explained 18% of variance in prolonged grief symptoms. Again, only kinship and age significantly added to the final model predicting prolonged grief symptoms. In the second step, rumination additionally explained another 21% of variance in prolonged grief symptoms (*β* = .38, *p* < .001). In the final step, worry explained another 2% of unique variance in prolonged grief symptoms (*β* = .20, *p* <. 001).

### 3.4 Mediation models of worry and rumination, avoidance processes, and depression and prolonged grief symptoms

A first multiple mediation analysis tested if the relation between rumination and depression symptoms was mediated by loss‐related and behavioural avoidance. The total direct effect of rumination on depression was significant (*β* = .43, *p* < .001). After controlling for the indirect effects of the mediators (*β* = .30, 95% CI [0.23, 0.36]), the direct effect became smaller but was still significant (*β* = .13, *p* < .001), indicating partial mediation. Behavioural avoidance was a significant mediator of the relationship between rumination and depression symptoms (*β* = .32, 95% CI [0.25, 0.38]), whereas loss‐related avoidance was not (*β* = −.02, 95% CI [−0.05, 0.01]). The mediation effect size was .69.

A second multiple mediation model assessed if the effect of worry on depression symptoms was mediated by loss‐related and behavioural avoidance. The total direct effect of worry on depression symptoms was significant (*β* = .42, *p* < .001). After controlling for the indirect effect of loss‐related and behavioural avoidance (*β* = .29, 95% CI [0.22, 0.35]), the direct effect of worry on depression remained significant (*β* = .13, *p* < .001), signifying a partial mediation. Again, behavioural avoidance was a significant mediator of the relation between worry and depression (*β* = .30, 95% CI [0.23, 0.36]), but loss‐related avoidance was not (*β* = −.01, 95% CI [−0.04, 0.01]). The mediation effect size was .67.

In a third multiple mediation model, we tested if the relation between rumination and prolonged grief symptoms was mediated by loss‐related and behavioural avoidance. The total direct effect of rumination on prolonged symptoms was significant (*β* = .50, *p* < .001). After controlling for the indirect effect of loss‐related and behavioural avoidance (*β* = .29, 95% CI [0.23, 0.35]), the association between rumination on prolonged grief was less strong but still significant (*β* = .21, *p* < .001), demonstrating partial mediation. Behavioural and loss‐related avoidance both mediated the effect significantly (*β* = .22, 95% CI [0.17, 0.27]; *β* = .07, 95% CI [0.04, 0.11], respectively). The effect size of the indirect effect was .58, of which .15 could be attributed to both loss‐related avoidance and .43 to behavioural avoidance.

In the fourth mediation analysis, we tested if the relation between worry and prolonged grief symptoms is mediated by loss‐related and behavioural avoidance. Again, we found a partial mediation. The total direct effect of worry on prolonged symptoms was significant (*β* = .42, *p* < .001) and remained significant after accounting for indirect effects (*β* = .14, *p* < .001). The total indirect effect (*β* = .28, 95% CI [0.22, 0.34]) was composed of significant mediation through behavioural avoidance (*β* = .21, 95% CI [0.16, 0.26]) and loss‐related avoidance (*β* = .07, 95% CI [0.04, 0.10]). The relative effect size of the mediation was .67, of which .16 through loss‐related avoidance and .51 through behavioural avoidance.

## 4 DISCUSSION

The present study sought to clarify the role of repetitive thought in psychological adaptation to loss. The first aim of this study was to establish the relative association of rumination and worry with depression and prolonged grief symptoms. In line with our expectations, rumination and worry were both positively associated with depression and prolonged grief symptoms. Worry was less strongly correlated with prolonged grief symptoms (but not depression symptoms) than rumination. Worry was further associated with prolonged grief and depression symptoms even after controlling for rumination and relevant demographic and loss‐related variables. The second aim of this study was to examine two putative working mechanisms in the relationships between these repetitive thought styles and mental health problems. Partially confirming predictions, rumination and worry were both associated with depressive symptoms, and these associations were partially mediated by behavioural avoidance but not by loss‐related avoidance. In line with our hypotheses, the associations between worry and rumination and prolonged grief symptoms were partially explained by loss‐related avoidance and behavioural avoidance.

The finding that rumination and worry were both uniquely associated with depression and prolonged grief symptoms in multivariate regression models confirms the importance of both repetitive thought styles in adaptation to bereavement. Results are in line with a larger research body on the effects of rumination following bereavement (for a review, see Eisma & Stroebe, 2017) and with three prior studies on worry and post‐loss psychopathology (Boelen, 2010; Boelen et al., 2016; Eisma et al., 2017). We also confirmed earlier findings suggesting that rumination may be more strongly associated with post‐loss mental health outcomes than worry (Boelen et al., 2016). Our first empirical test of these associations suggested that rumination may play a larger role in prolonged grief responses than in depressive reactions to loss. Nevertheless, on balance, both processes appear of some importance in adaptation to loss and should thus both be targeted in repetitive thought focused treatments for severe grief reactions (e.g., Wenn et al., 2019). It should be noted here that the size of associations between these cognitive processes and emotional problems may differ depending on the type of rumination under investigation. For example, we used a measure of depressive rumination in the present study, the brooding subscale of the RRS, but grief‐specific rumination about the causes and consequences of a loss is concurrently and longitudinally more strongly associated with post‐loss mental health problems than depressive rumination (Eisma & Stroebe, 2017).

Relationships between rumination and worry and depression symptoms were both mediated by behavioural avoidance but not by loss‐related avoidance. The findings on rumination are in line with the results from a longitudinal mediation study by Eisma et al. (2013). To the best of our knowledge, our study is the first to demonstrate this effect for worry. This could imply that rumination and worry both take up attention and time that could be used to undertake social, recreational, and occupational activities, and provide complementing rationales for such behavioural withdrawal. That is, rumination may strengthen convictions that the current situation is hopeless and nothing can be done to change it (Nolen‐Hoeksema et al., 2008), whereas worry can inflate perceptions of the probability and consequences of aversive outcomes of future events. As a consequence of such behavioural withdrawal, bereaved persons could experience fewer opportunities to disconfirm negative cognitions about the world and lift their negative mood.

Both worry and rumination had indirect effects via loss‐related avoidance and behavioural avoidance on prolonged grief symptoms. This suggest that worry and rumination may both act as cognitive avoidance strategies, for example, by suppressing more threatening loss‐related thoughts and memories, thereby prolonging grief reactions (e.g., Boelen et al., 2006; Eisma et al., 2013). However, both repetitive thought styles may also contribute to withdrawal from activities. Therefore, bereaved people may have fewer opportunities to engage in rewarding activities that could disconfirm negative cognitions, and expose themselves less to situations in which the absence of the deceased is felt strongly, thereby prolonging grief reactions.

While the working mechanisms of worry have not been previously investigated in bereaved samples, the findings on mediation effects of avoidance processes between rumination and prolonged grief are different from a longitudinal study from Eisma et al. (2013). In that three‐wave study, loss‐related avoidance and behavioural avoidance did not longitudinally mediate relationship between rumination and prolonged grief symptoms in bereaved persons (but experiential avoidance and thought suppression were significant mediators). These differences in outcomes can be due to various methodological issues. For example, in Eisma et al.'s (2013) study, baseline symptoms were controlled for in longitudinal analyses, leaving substantially less variance to be accounted for by avoidance processes in the multiple mediator model. Therefore, despite moderate to high positive associations between all avoidance processes, rumination and prolonged grief levels, only the strongest mediators yielded significant indirect effects in that study. Moreover, Eisma et al. (2013) examined four different types of avoidance, which showed some conceptual overlap, whereas the present study only used two conceptually distinct avoidance processes as mediators. If mediators partially explain the same variance, only those processes relating most strongly to the dependent and independent variable will be significant mediators. For instance, if loss‐related avoidance partly takes place through thought suppression, the mediation effect of thought suppression could render the effect of loss‐related avoidance insignificant in a multiple mediator model. To further clarify the role of rumination and worry and post‐loss adaptation, and the link between these cognitive processes and avoidance tendencies, we recommend intensive multivariate longitudinal studies, including diary and ecological momentary assessment research (for an example in a nonbereaved sample, see Dickson, Ciesla, & Reilly, 2012).

Clinically, our findings suggest that targeting loss‐related and behavioural avoidance in treatment is effective in reducing repetitive thought and loss‐related distress. Specifically, results align with studies demonstrating that exposure (which aims to reduce avoidance of loss‐related memories, objects, or situations) and behavioural activation (which aims to increase the number of rewarding and valued activities) reduce rumination in treatments for prolonged grief (e.g., Eisma, Boelen, et al., 2015; Papa, Rummel, Garrison‐Diehn, & Sewell, 2013). Other trials in nonbereaved samples suggest that the effects of these treatments may also effectively reduce worry (e.g., Chen et al., 2013; Hoyer et al., 2009). Future controlled trials and laboratory investigations are recommended to establish if such effects also hold in people experiencing high levels of worry and post‐loss psychopathology.

Although the present investigation in a large community sample of recently bereaved people offers unique and clinically relevant insights, some limitations are notable. First, as is common in voluntary bereavement research, higher educated women were overrepresented in our sample. Future research could aim to establish if the present results hold in a sample with more men and lower education levels. Second, our cross‐sectional design precludes drawing conclusions about temporal precedence and causality between the variables under investigation. Relatedly, it was not possible to evaluate to what extent in particular depressive symptomatology was already present before bereavement, or how our independent variables and mediators may have exacerbated such existing mental health problems. Third, our survey methodology comes with specific drawbacks, including various biases, such as recency bias (e.g., scale scores on behavioural avoidance may be influenced to a higher degree by recent events as these are more easily remembered). Therefore, we recommend further studies of the interrelations between repetitive thought styles and avoidance after bereavement using a variety of different methods that overcome these limitations such as experiments (e.g., Giorgio et al., 2010) and experience sampling (e.g., Dickson et al., 2012). Fourth, this study employed a measure of depressive rumination to assess maladaptive repetitive thought about causes and consequences of dysphoric feelings. The related construct of grief rumination, repetitive thought about causes and consequences of a loss, has been argued and empirically demonstrated to play a more central role than depressive rumination in psychological adaptation to bereavement (for a review, see Eisma & Stroebe, 2017). The continued use of measures to assess grief‐specific rumination, such as the well‐validated Utrecht Grief Rumination Scale (Eisma et al., 2014), is therefore warranted in future research on repetitive thought in bereavement.

In conclusion, the present study confirms the importance of both rumination and worry in coming to terms with bereavement and suggests that both processes may hamper the recovery because they are linked with specific avoidance processes. These findings are in line with prior research on the effects and working mechanisms of rumination yet also uniquely demonstrate the role and potential function of worry in the grieving process. Future research should aim to replicate and extend these findings with more advanced designs and methodology, with the ultimate goal to improve clinical practice for severely distressed bereaved people who engage in (chronic) repetitive thought.

## CONFLICT OF INTEREST

None.
